# Supplementary material for: Regulation of ammonium acquisition and use in Oryza longistaminata ramets under nitrogen source heterogeneity
Source: Plant Physiol. 2022 Feb 4;188(4):2364–76. doi: 10.1093/plphys/kiac025 (PMC8968255; doi:10.1093/plphys/kiac025)
Supplement: kiac025_Supplementary_Data [file kiac025_supplementary_data.zip › Supplemental Fig S1 to S7.pdf]

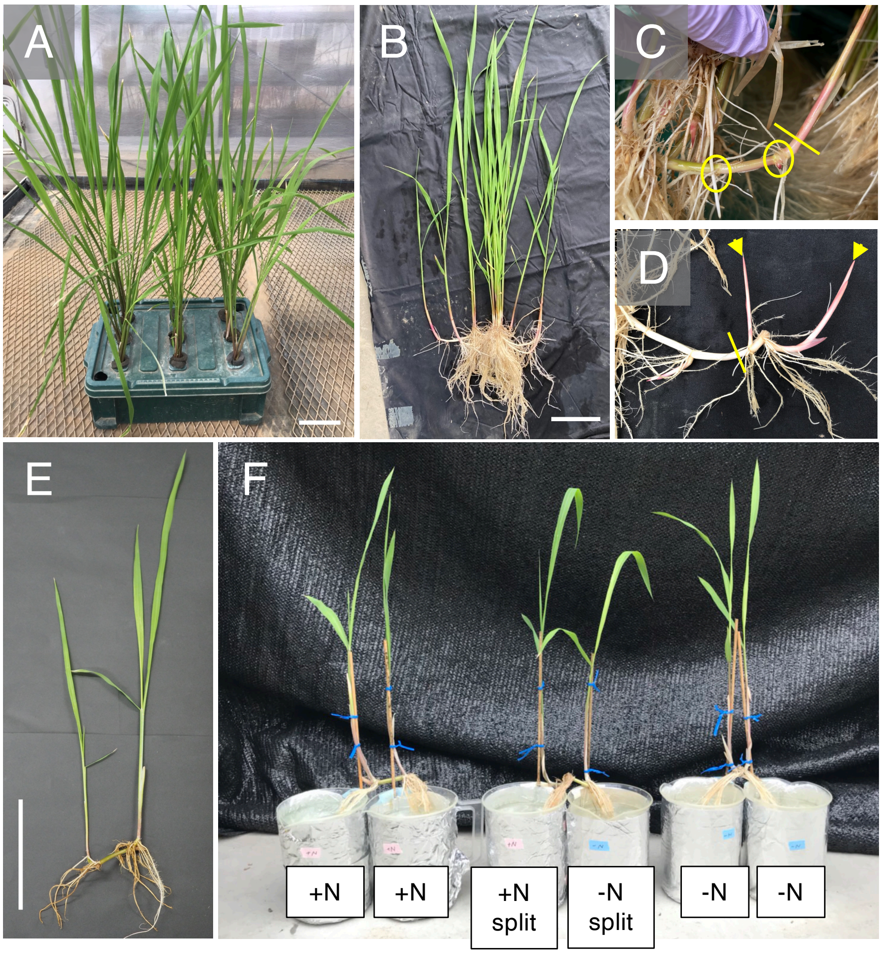


**Supplemental Figure S1.** The split hydroponic experimental system for *O. longistaminata* ramet pairs. A, *O. longistaminata* hydroponic culture. The plants were maintained in a 10 L-container. Scale bar, 10 cm. B, A clonal colony of *O. longistaminata*. Scale bar, 10 cm. C, A rhizome grown in the hydroponic culture. Ovals identify adjacent nodes. The tip of a primary rhizome was cut at the yellow line to induce the outgrowth of secondary rhizomes. D, Growth of secondary rhizomes at adjacent nodes (arrowheads). The pair was excised from the parental colony at the yellow line for further growth. E, A ramet pair grown from adjacent rhizome nodes. Scale bar, 15 cm. F, Split treatments with different nitrogen conditions. Each pot contains 1 L of nutrient solution.

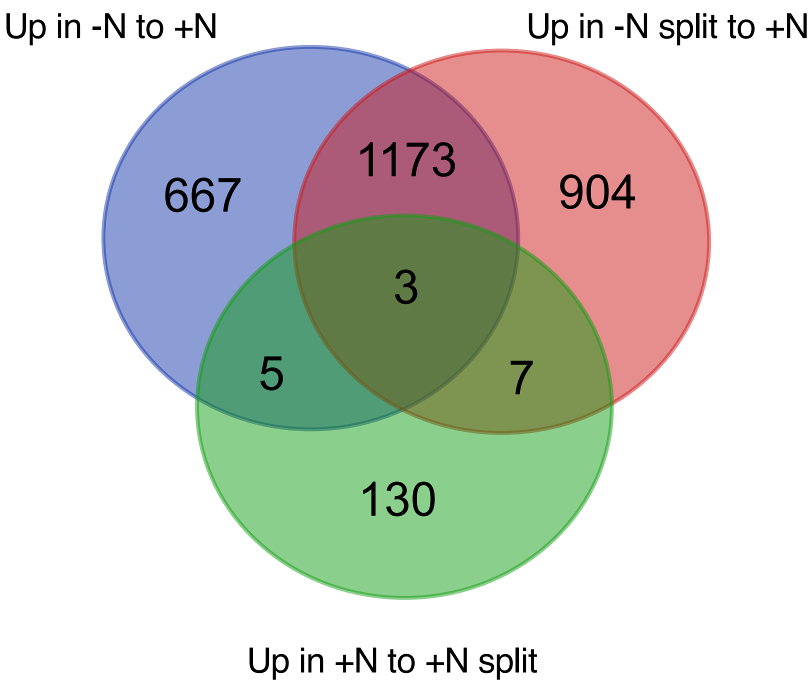


**Supplemental Figure S2.** A Venn diagram showing the overlap among genes up-regulated in the -N treatment compared to the +N treatment (Up in -N to +N), those up-regulated in the -N split treatment compared to +N treatment (Up in -N split to +N), and those up-regulated in the +N treatment compared to +N split treatment (Up in +N to +N split).

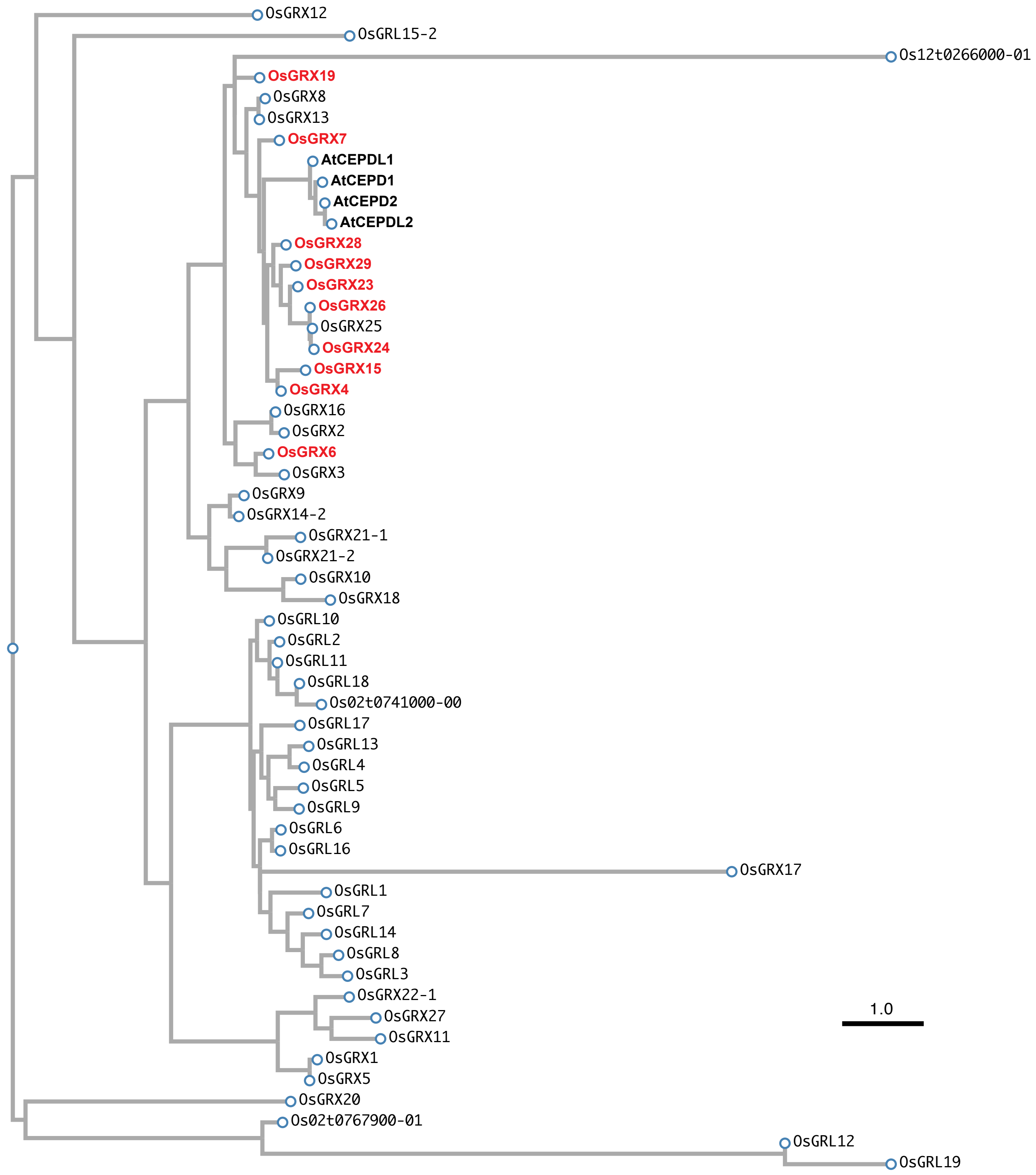


**Supplemental Figure S3.** Phylogenetic analysis of Arabidopsis CEPDs (AtCEPD1, AtCEPD2, AtCEDL1, AtCEPDL2) and *O. sativa* glutaredoxin family proteins, GRX and GRL. Alignment and phylogenetic reconstructions were performed using the function “build” of ETE3 v3.1.1 (Huerta-Cepas et al., 2016) as implemented on the GenomeNet (https://www.genome.jp/tools/ete/). An alignment of the amino acid sequences used for this analysis is shown in Supplemental Figure S4. Scale bar, substitution per site.

**Huerta-Cepas J, Serra F, Bork P** (2016) ETE 3: reconstruction, analysis, and visualization of phylogenomic data. Mol Biol Evol **33**: 1635–1638

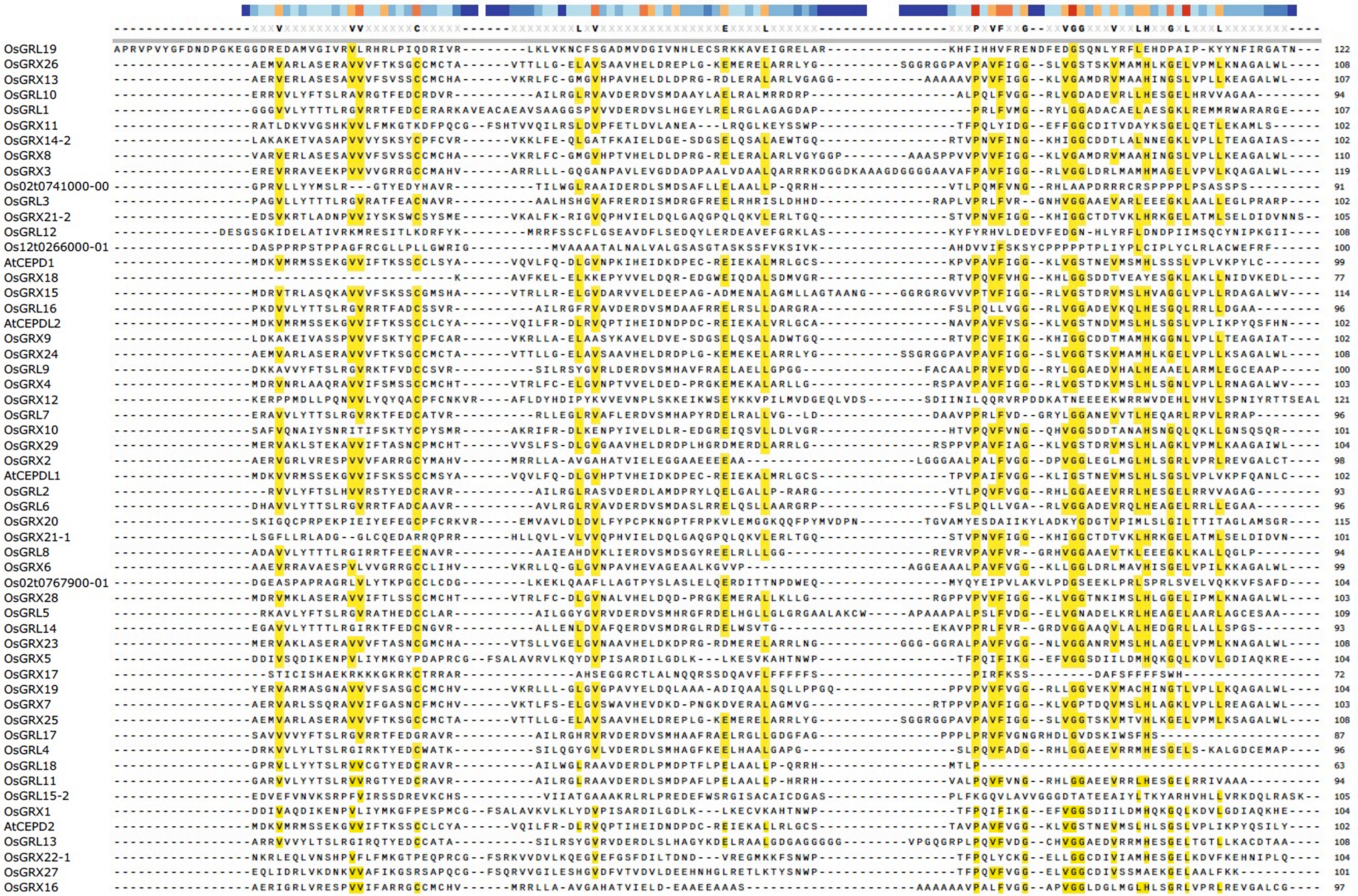

**Supplemental Figure S4.** An alignment of the amino acid sequences used for the phylogenetic analysis in Supplemental Figure S3. AtCEPD1 (At1g06830), AtCEPD2 (At2g47880), AtCEPDL1 (At2g30540), AtCEPDL2 (At3g62960), OsGRX1 (Os01t0174900), OsGRX2 (Os01t0194600), OsGRX3 (Os01t0241400), OsGRX4 (Os01t0368900), OsGRX5 (Os01t0530400), OsGRX6 (Os01t0667900), OsGRX7 (Os01t0936000), OsGRX8 (Os02t0512400), OsGRX9 (Os02t0618100), OsGRX10 (Os02t0646400), OsGRX11 (Os03t0851200), OsGRX12 (Os04t0244400), OsGRX13 (Os04t0393500), OsGRX14 (Os04t0508300), OsGRX15 (Os05t0149950), OsGRX16 (Os05t0198200), OsGRX17 (Os05t0563900), OsGRX18 (Os06t0659500), OsGRX19 (Os07t0151100), OsGRX20 (Os08t0558200), OsGRX21-1 (Os08t0565800-01), OsGRX21-2 (Os08t0565800-02), OsGRX22 (Os10t0500700), OsGRX23 (Os11t0655900), OsGRX24 (Os11t0656000), OsGRX25 (Os11t0656400), OsGRX26 (Os11t0656700), OsGRX27 (Os12t0175500), OsGRX28 (Os12t0538600), OsGRX29 (Os12t0538700), OsGRL1 (Os01t0235900), OsGRL2 (Os01t0829400), OsGRL3 (Os02t0102000), OsGRL4 (Os02t0748800), OsGRL5 (Os03t0170800), OsGRL6 (Os03t0356400), OsGRL7 (Os03t0648800), OsGRL8 (Os04t0412800), OsGRL9 (Os04t0641300), OsGRL10 (Os05t0353600), OsGRL11 (Os05t0471350), OsGRL12 (Os06t0224200), OsGRL13 (Os06t0226100), OsGRL14 (Os07t0159900), OsGRL15 (Os07t0657900), OsGRL16 (Os07t0659900), OsGRL17 (Os08t0171333), OsGRL18 (Os08t0554699), OsGRL19 (Os10t0482900), Os02t0741000-00, Os02t0767900-01, Os12t0266000-01. Highly conserved amino acid residues are highlighted in yellow.

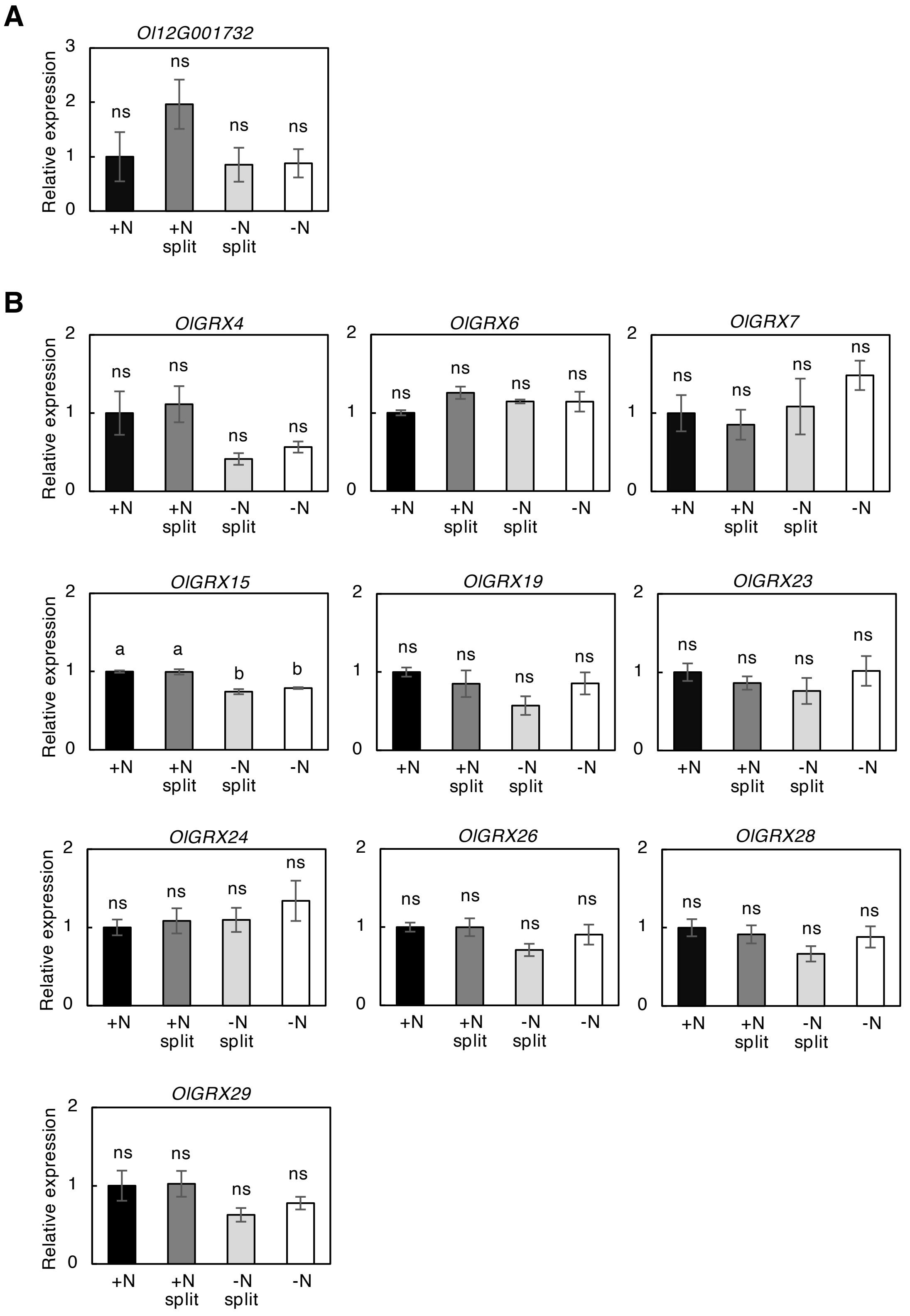


**Supplemental Figure S5.** Expression patterns of *Ol12G001732* and *OlGRXs*. A, Expression pattern of *Ol12G001732,* the closest homolog of Arabidopsis *CEPR1.* B, Expression pattern of *OlGRXs,* homologs of Arabidopsis *CEPDs*, in the shoots of ramet pairs after a 24-h split treatment. The expression level of each gene normalized by *TBC* is expressed relative to that of the +N treatment defined as 1. Error bars represent the SE of values for biological replicates (n = 3 or 4). Different lowercase letters at the top of each column denote statistically significant differences by Tukey's honestly significant difference test (HSD) ($p < 0.05$). ns, not significant.

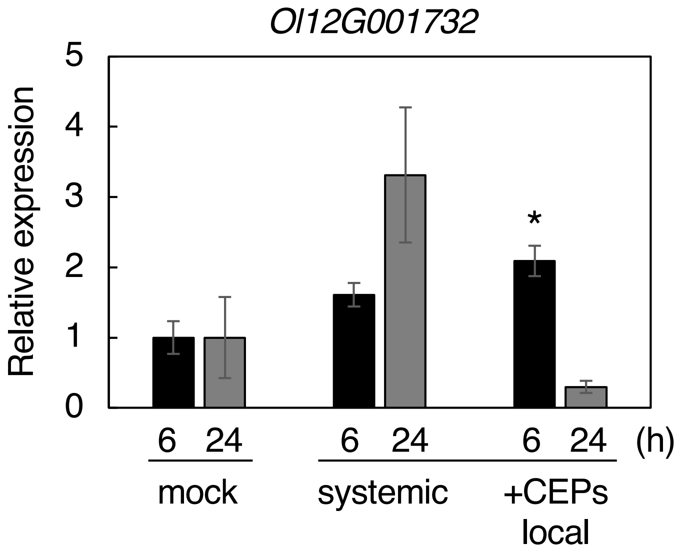


**Supplemental Figure S6.** Expression of *Ol12G001732* in ramet shoots in response to exogenously supplied CEP1 peptides. The CEP1 peptide mix was applied to the roots of one ramet pair. Gene expression was measured in shoots from the local (+CEP local) and systemic side (systemic) after 6 and 24 h treatment. The expression level of *Ol12G001732*, normalized by *TBC*, is expressed relative to that of the +N mock treatment defined as 1. Error bars represent the SE of values for biological replicates (n = 3 or 4). $^{*}p < 0.05$ (Student's t-test) compared to the corresponding mock treatment.

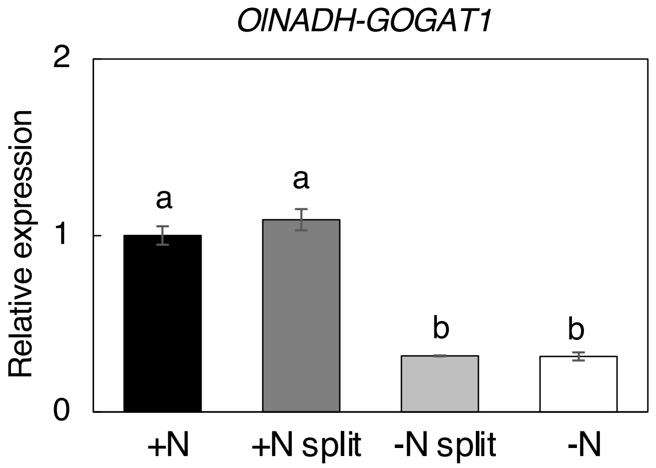


**Supplemental Figure S7.** Transcript abundance of *OlNADH-GOGAT1* in the roots of ramet pairs after a 24-h split treatment as measured by RT-qPCR. The expression level of each gene, normalized by *TBC*, is expressed relative to that of the +N treatment defined as 1. Error bars represent SE of values for biological replicates (n = 3 or 4). Different lowercase letters at the top of each column denote statistically significant differences by Tukey's honestly significant difference test (HSD) ($p < 0.05$).
